# Design and Fabrication of a Novel MEMS Relay with Low Actuation Voltage

**DOI:** 10.3390/mi11020171

**Published:** 2020-02-07

**Authors:** Hao Li, Yong Ruan, Zheng You, Zhiqiang Song

**Affiliations:** 1 State Key Laboratory of Precision Measurement Technology and Instruments, Department of Precision Instrument, Tsinghua University, Haidian District, Beijing 100084, China; lihaothu@tsinghua.edu.cn (H.L.); yz-dpi@mail.tsinghua.edu.cn (Z.Y.); 2 MEMS Institute of Zibo National High-Tech Industrial Development Zone, Zibo 255000, China; songzqmic@163.com

**Keywords:** micro-electro mechanical system (MEMS) relay, cantilever, low actuation voltage

## Abstract

Compared with conventional solid-state relays, micro-electro mechanical system (MEMS) relays have the advantages of high isolation, low contact resistance, low power consumption, and abrupt switching characteristics. Nevertheless, the widespread application of MEMS relays has been limited due to the issue of the conflict between low actuation voltages and high device performance. This article presents a novel cantilever MEMS relay with an embedded contact electrode which helps to achieve a low actuation voltage (below 8 V) and high restoring force simultaneously. Meanwhile, the contact resistance is as low as around 0.4 Ω and the reliability is verified. To thoroughly investigate and analyze the novel cantilever MEMS relay, a static theoretical model of the structure was developed. Based on the model, the cantilever MEMS relay was designed and optimized. Then, the relays were fabricated by the bulk-silicon micromachining process based on the silicon–glass anodic bonding technology. Finally, the switching performance of the novel cantilever MEMS relay was measured. Experimental results demonstrate that the proposed MEMS relay has a low actuation voltage below 8 V and high performance, which is in good agreement with the simulation results, and shows significant advantages when compared with previous reports. Therefore, the proposed MEMS relay with an embedded contact electrode is promising in practical applications.

## 1. Introduction

Micro-electro mechanical system (MEMS) relays have the potential to be applied in space technology, communication, and automatic devices [1]. Compared to conventional solid-state relays, MEMS relays have the advantages of high isolation, low on-resistance, low power consumption, and abrupt switching characteristics [2]. Besides, they are reliable and inexpensive to facilitate packaging and system integration as they can be batch manufactured like solid-state relays [3,4].

Among various driving methods for MEMS relays, electrostatic actuation is mostly utilized owing to the advantages of low power and easy fabrication [5]. Nevertheless, electrostatic MEMS relays have the issue of the conflict between low actuation voltages and good device performance. In general, the low actuation voltage implies a low contact force or low restoring force. The former leads to a high contact resistance [6], while the latter contributes to an easily irreversible stiction [7]. Different ideas have been proposed to reduce the actuation voltage without lowing the contact force or restoring force. For example, novel spring structures have been exploited to reduce the actuation voltage [8,9], but the structural and fabrication complexity is increased. Another method to lower the driving voltage requires an additional pre-charged electrode [10,11].

Component reliability is another issue for electrostatic MEMS relays. While stiction is a major cause for low reliability, numerous studies have dedicated efforts to enhance restoring force to overcome stiction. Oberhammer has designed a novel mechanism to acquire a large active opening force, but the contact force is decreased [12]. Increasing the electrode area is another mechanical approach to enhance restoring force that does not sacrifice the contact force or actuation voltage. However, it requires a bigger size [13]. Other researches utilize special active anti-stiction mechanisms to provide extra restoring force [7,14,15], but they increase the structural and fabrication complexity.

In this article, we present a novel cantilever MEMS relay with an embedded contact electrode. This embedded contact electrode structure helps to achieve a low actuation voltage (below 8 V). Meanwhile, the contact resistance is as low as around 0.4 Ω and the switching-on time and switching-off time are lower than 100 μS. To thoroughly investigate and analyze the novel cantilever MEMS relay, a static theoretical model of the structure was developed. Based on the model, the cantilever MEMS relay was designed and optimized. Then, the relays were fabricated by the bulk-silicon micromachining process based on the silicon–glass anodic bonding technology. Finally, the switching performance of the novel cantilever MEMS relay was measured. The experimental results demonstrate that the MEMS relay has a low actuation voltage and high performance.

## 2. Design of the Cantilever Micro-Electro Mechanical System (MEMS) Relay

**Figure 1:**
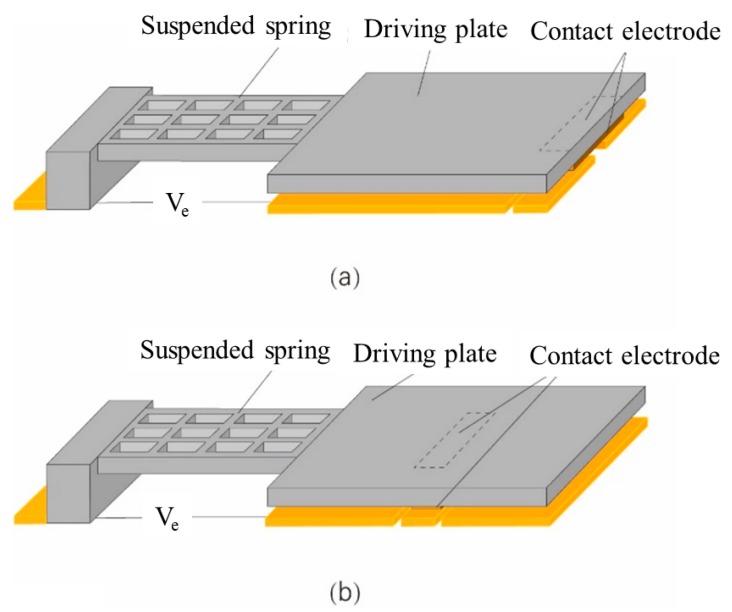
Schematic structure of the proposed micro-electro mechanical system (MEMS) relay for an individual element: (**a**) Contact electrode at the edge of the cantilever relay; (**b**) cantilever relay with an embedded contact electrode.

Figure 1 illustrates the proposed cantilever relay with an embedded contact electrode and a conventional cantilever switch. The designed MEMS relay is comprised of a hollow suspended spring, a driving plate, and a contact electrode. The hollow suspended spring lowers the actuation voltage and enhances the device stability considerably, which has been demonstrated in our earlier work [4].

## 3. Modeling and Simulation

### 3.1. Static Modeling

**Figure 2:**
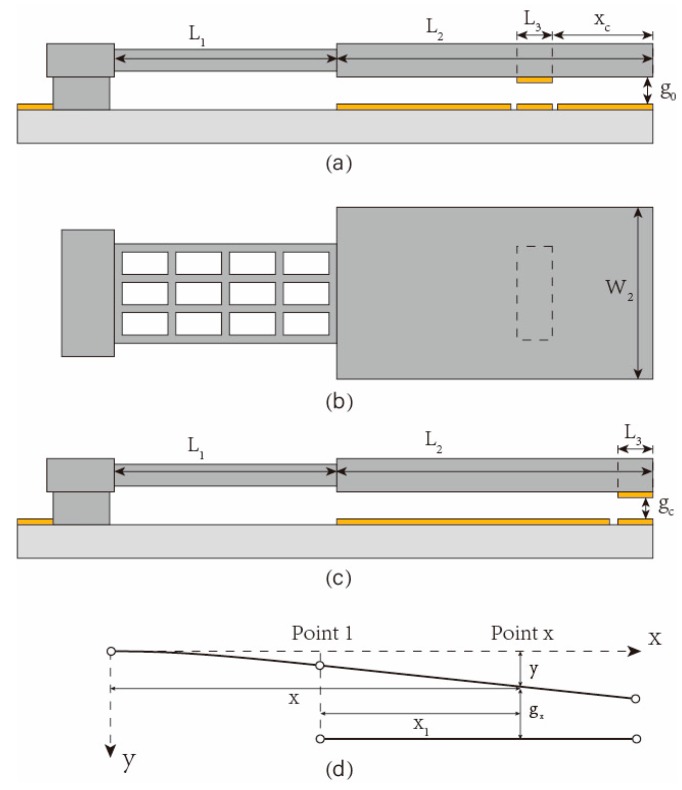
The equivalent parameters of the proposed MEMS relay: (**a**–**c**) Dimension marking of geometric parameters for the cantilever; (**d**) establishment of coordinate for geometric parameters.

With the low stiffness of the suspended spring and high stiffness of the driving plate, the driving plate can be regarded as a rigid body. As shown in Figure 2d, Point 1 is the joint between the spring and the plate, ω_1_ is its deflection, and *θ*_1_ is its rotation angle. The deflection at Point x can be derived:

(1) $$y=\omega_{1}+\theta_{1}x_{1},$$

where *x*_1_ designates the location of Point *x* as shown in Figure 2d. Therefore, the magnitude of the electrostatic force at Point *x* can be determined (Point *x* is not at the contact electrode):

(2) $$q_{x}=\varepsilon_{0}\frac{V_{e}{}^{2}}{2{\left(g_{0}-y\right)}^{2}}W_{2},$$

where *ε*_0_ is the permittivity of air, *g*_0_ is the original air gap between the cantilever beam and the gate electrode, *V*_e_ is the driving voltage, and *W*_2_ is the plate width. *M_qx_* can be derived as:

(3) $$M_{q_{x}}=\varepsilon_{0}\frac{V_{e}{}^{2}}{2{\left(g_{0}-y\right)}^{2}}W_{2}\cdot x_{1},$$

The electrostatic forces along the plate are equivalent to a concentrated load at Point 1, which can be determined by integrating Equations (2) and (3):

(4) $$F_{e}=\int_{0}^{L_{2}}\varepsilon_{0}\frac{V_{e}{}^{2}}{2{\left(g_{0}-y\right)}^{2}}W_{2}dx_{1}-\int_{L_{2}-L_{3}-x_{c}}^{L_{2}-x_{c}}\varepsilon_{0}\frac{V_{e}{}^{2}}{2{\left(g_{0}-y\right)}^{2}}W_{2}dx_{1},$$

(5) $$M_{e}=\int_{0}^{L_{2}}\varepsilon_{0}\frac{V_{e}{}^{2}}{2{\left(g_{0}-y\right)}^{2}}W_{2}x_{1}dx_{1}-\int_{L_{2}-L_{3}-x_{c}}^{L_{2}-x_{c}}\varepsilon_{0}\frac{V_{e}{}^{2}}{2{\left(g_{0}-y\right)}^{2}}W_{2}x_{1}dx_{1},$$

where *x_c_* designates the location of the contact electrode as shown in Figure 2a. The deflection and rotation angle at Point 1 can be derived:

(6) $$\omega_{1}=\frac{F_{e}L_{1}{}^{3}}{3EI}+\frac{M_{e}L_{1}{}^{2}}{2EI},$$

(7) $$\theta_{1}=\frac{F_{e}L_{1}{}^{2}}{2EI}+\frac{M_{e}L_{1}}{EI},$$

where *E* and *I* are the Young’s modulus and the second moment of inertia of the suspended spring, and *L*_1_ is the spring length. When the driving voltage Ve is small, the deflection of the relay can be determined by numerical calculation by Equations (1)–(7). If there is no solution for Equations (1)–(7), *V*_e_ reaches the pull-in voltage.

The finite element model was established by COMSOL (version 5.4, COMSOL Co., Ltd., Stockholm, Sweden). The structural parameters of the traditional and proposed MEMS relay are listed in Table 1. We change the position of contact electrode and keep the other parameters unchanged to verify the effect of promoting the relay performance.

**Figure 3:**
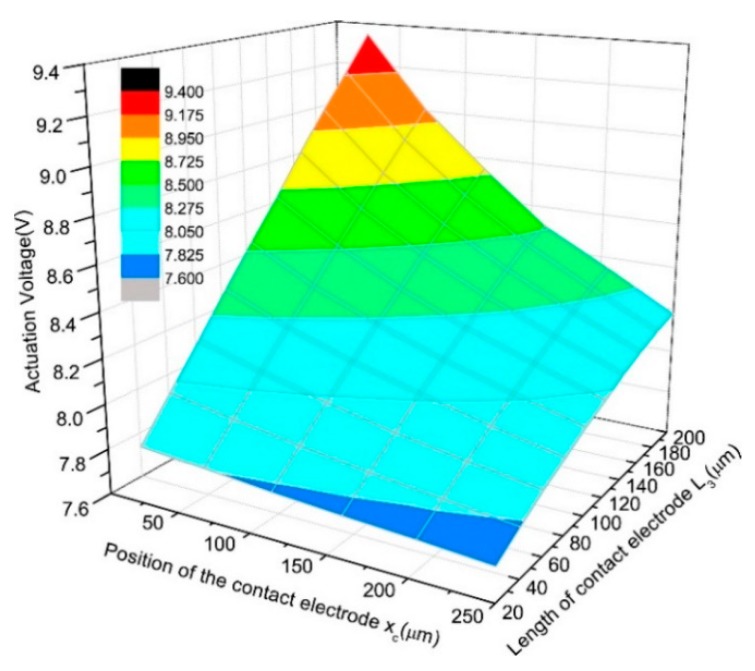
Simulated actuation voltage versus *x_c_* and *L*_3._

Figure 3 illustrates the simulated results of the actuation voltage. The simulation results show that the actuation voltage decreases with the contact electrode moving inside or becoming smaller. According to Figure 3, when the length of the contact electrode is 50 μm as designed, the actuation voltage drops from 8.02 V to 7.81 V when the contact electrode of the conventional relay moves 250 μm inside. The decrement is bigger if the area of the contact electrode becomes bigger.

**Figure 4:**
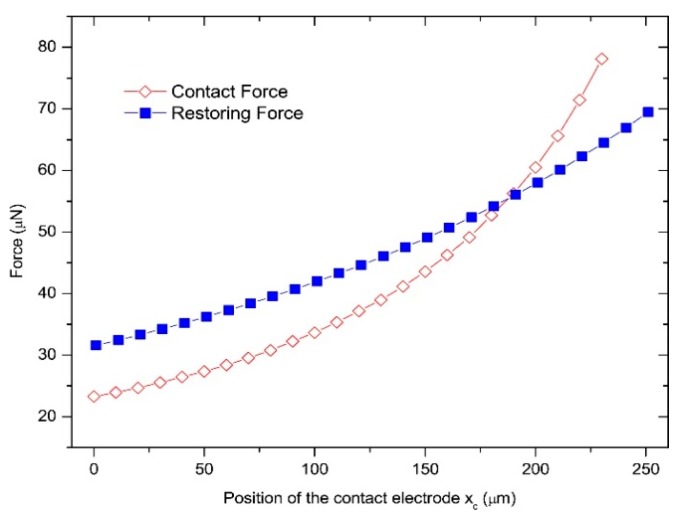
Simulated contact force and restoring force against *x_c_*_._

We also simulated the contact force and restoring force against the value of *x_c_*. We imposed the same actuation voltage of 9 V, and Figure 4 shows that the contact force increases dramatically with *x_c_* increasing. Then, we solved the minimal force imposed on the contact electrode that ensured the relay close. The minimal force, which is regarded as the restoring force when the relay keeps closed, also increases with *x_c_* increasing.

### 3.2. Dynamic Modeling

The dynamic model is established using a similar way which has been introduced in earlier work. The dynamic Euler–Bernoulli beam equation can describe the transient response of the cantilever relay:

(8) $$m\frac{\partial^{2}y}{\partial t^{2}}+D\frac{\partial y}{\partial t}+EI\frac{\partial^{4}y}{\partial x^{4}}=F_{e}-F_{c},$$

where *m* is the mass per unit length of the cantilever relay, *y*(*x*, *t*) is the downward deflection of the relay at time *t*, *D* is the damping factor, *EI* is the flexural rigidity, *F_e_* is the electrostatic force, and *Fc* is the contact force. The initial and boundary conditions are as follows:

(9) $$\left\{ \begin{array}{l} y(x,0)=0,\\ \frac{\partial y(x,0)}{\partial t}=0, \end{array}\right.$$

(10) $$\left\{ \begin{array}{l} y(0,t)=0,\;\frac{\partial y(0,t)}{\partial x}=0,\\ \frac{\partial^{2}y(l,t)}{\partial x^{2}}=0. \end{array}\right.$$

Since the suspended spring of the relay is hollowed, the air damping of the spring can be ignored. It is assumed that the relay operates in an air medium, then the air damping of the driving plate can be simplified as:

(11) $$D=KW_{2}\frac{\mu }{1+9.638{\left(\lambda_{0}/g_{0}\right)}^{1.159}}\frac{L_{2}{}^{2}}{{\left(g_{0}-y\right)}^{3}}.$$

where *K* is the flow coefficient, which is 0.013 in our design; μ is the air damping coefficient, and *λ*_0_ is the mean free path of the air molecules (≈64 nm).

The electrostatic force is:

(12) $$F_{e}=\varepsilon_{0}\frac{V_{e}{}^{2}}{2{\left(g_{0}-y\right)}^{2}}W_{2}.$$

The contact force can be approximated by a linear spring model:

(13) $$F_{c}=k_{c}(y-g_{c})\cdot H(y-g_{c}),x\in [L_{1}+L_{2}-L_{3}-x_{c},L_{1}+L_{2}-x_{c}],$$

where the Heaviside function *H*(*y* − *g_c_*) ensures that the force is only applied when the relay makes contact. The spring constant kc takes an empirical value, ensuring the contact deformation is small.

**Figure 5:**
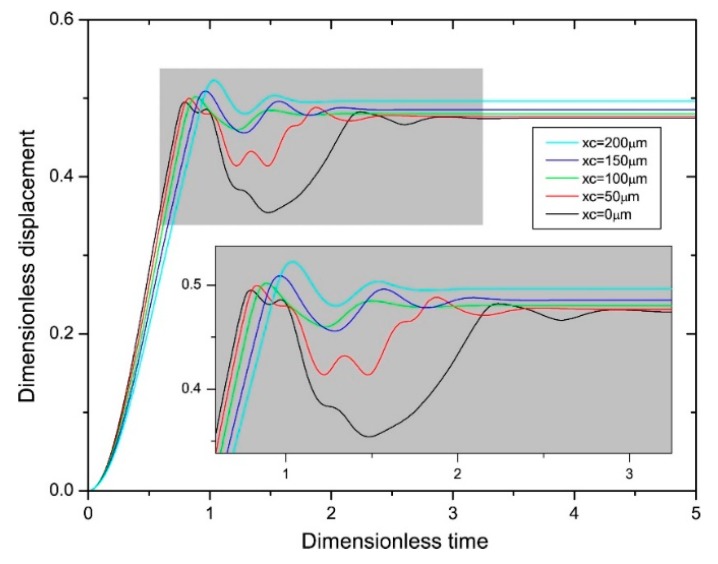
The simulated dynamic responses of the conventional and proposed MEMS relay of different contact electrode positions.

The dynamic model was simulated by COMSOL. Figure 5 illustrates the simulated dynamic responses of the conventional and proposed MEMS relay of different contact electrode positions. Being imposed the same driving voltage of 15 V, the dynamic bounce and switching time are both suppressed when the contact electrode is moving inside. Particularly, the switching time of the conventional relay (*x_c_* = 0 μm) is about 2.8 time units, while the time is diminished to 1.8 time units when *x_c_* is 200 μm as Figure 5 shows. When the contact electrode is moving inside, it is surrounded by the driving plate. Thus, the electrostatic force around the contact electrode restrains the contact bounces, which further reduces the switching time. As is shown in Figure 5, the optimal *x_c_* is about 100 μm, which has the minimum contact bounce time.

## 4. Fabrication for MEMS Relay

**Figure 6:**
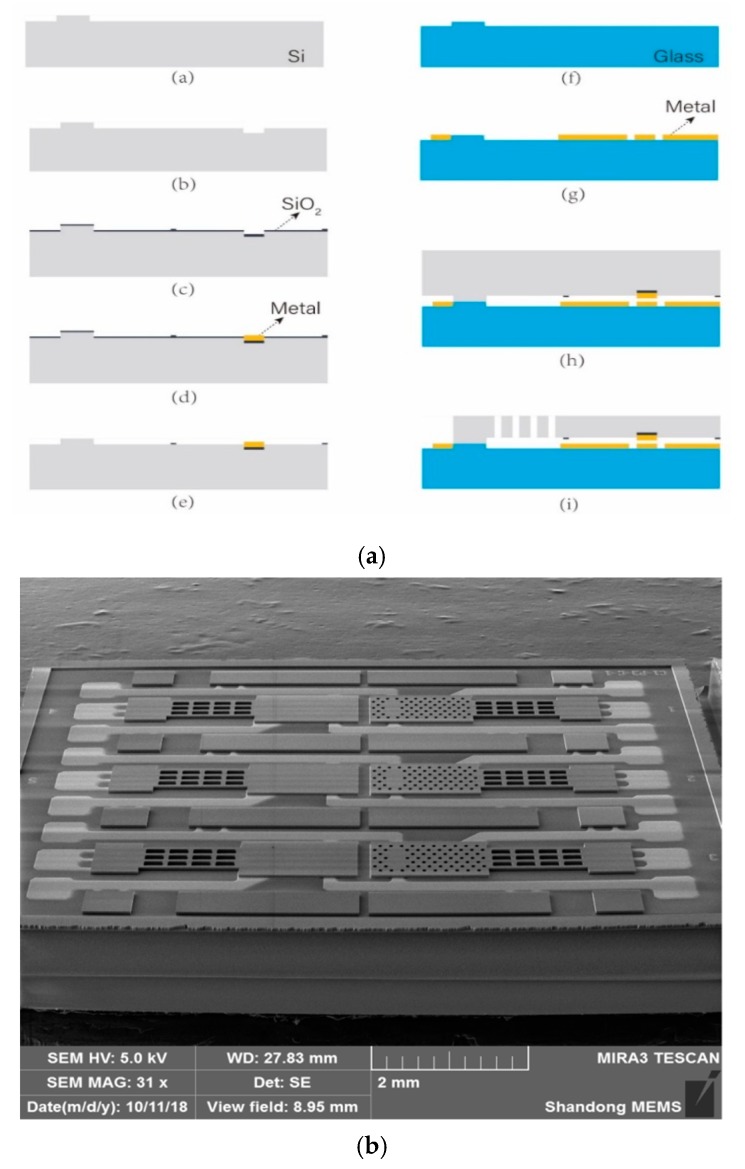
Microfabrication process and SEM image of the MEMS relay array. (**a**) Microfabrication process of the proposed MEMS relay array; (**b**) SEM image of the MEMS relay array.

The proposed cantilever relays with embedded contact electrodes were fabricated using bulk-silicon techniques based on the silicon–glass anodic bonding to form and pattern the mechanical and actuation structures. The fabrication process is summarized in Figure 6. First, a silicon wafer with a polished surface was patterned and etched to a depth of 1.5 μm. Next, another step of 1.0 μm was etched to form the dimple for the contact electrode. Then, a 4000 Å SiO_2_ insulating layer was deposited on the silicon device layer, and then the unexposed region of the SiO2 layer was etched to a depth of 2000 Å. Next, a Cr (400 Å)/Au (10,700 Å)/Pt (200 Å)/Au (1000 Å)/Pt (200 Å)/Au (500 Å) metal layer (13,000 Å in total) was sputtered and patterned by a lift-off process. Then, the SiO2 layer was etched to a depth of 2000 Å to remove the unexposed region. On the other side, a Pyrex 7740 glass wafer was etched 12,500 Å step. Then, the same metal layer as in Step (d) was sputtered to form the electrodes and leads. Next, the silicon layer after Step (e) was anodically bonded to the glass substrate after Step (g) and thinned to 22 μm. Finally, the device layer was etched by ICP (Inductively Coupled Plasma) to release the relay structures.

## 5. Experimental Results and Discussion

### 5.1. Test Experiment Platform

**Figure 7:**
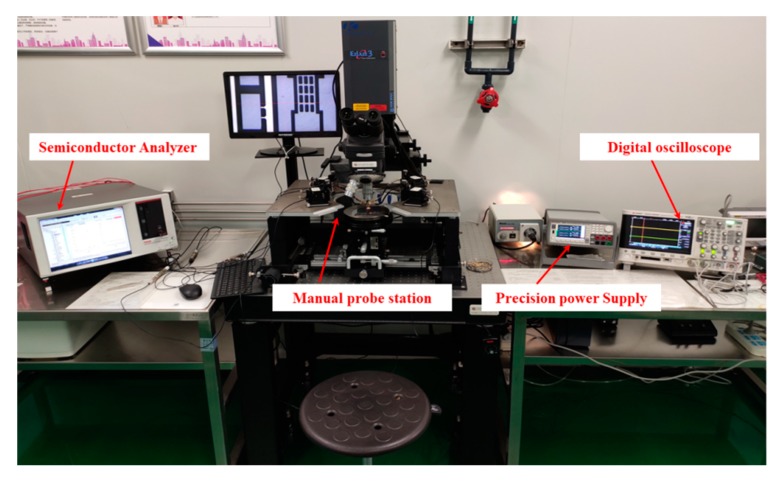
Experiment platform for electrical performance.

Figure 7 shows the experimental platform for electrical performance of the proposed MEMS relay. It contains a semiconductor analyzer (4200-SCS, Tektronix Inc., Johnston, OH, USA), a manual probe station (M8, Semiprobe Inc., Winooski, VT, USA), a precision power supply (B2902A, Agilent Technologies Inc., Santa Clara, CA, USA), and a digital oscilloscope (Agilent Technologies Inc., Santa Clara, CA, USA). The drive and load terminals of the proposed relay are connected to the manual probe station. The semiconductor analyzer applies a scanning voltage of 0 to 15 V to the drive terminals and records the hysteresis loop of pull-in and pull-off voltage. The digital oscilloscope is used to record the switching-on time, switching-off time, and switching state when the pull-in voltage and pull-off voltage are attained. A precision power supply and semiconductor analyzer are used to measure the contact resistance.

### 5.2. Pull-In and Pull-Off Voltage

**Figure 8:**
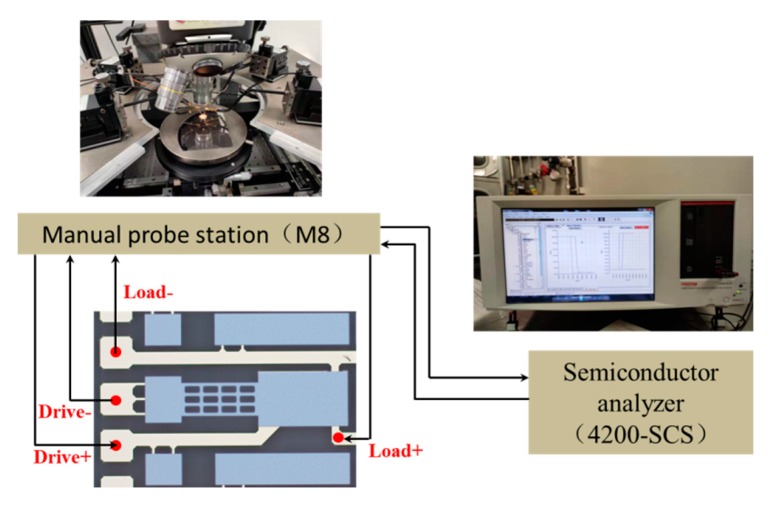
The setup schematic for measuring the actuation voltage.

The proposed MEMS relay uses voltage as the excitation quantity, and the actuation voltage refers to the corresponding voltage value that makes the MEMS relay act. According to the characteristics of MEMS relay, it includes the minimum action voltage, which is also called pull-in voltage, and the maximum release voltage, which is called pull-off voltage. Figure 8 shows the setup schematic for measuring the actuation voltage.

**Figure 9:**
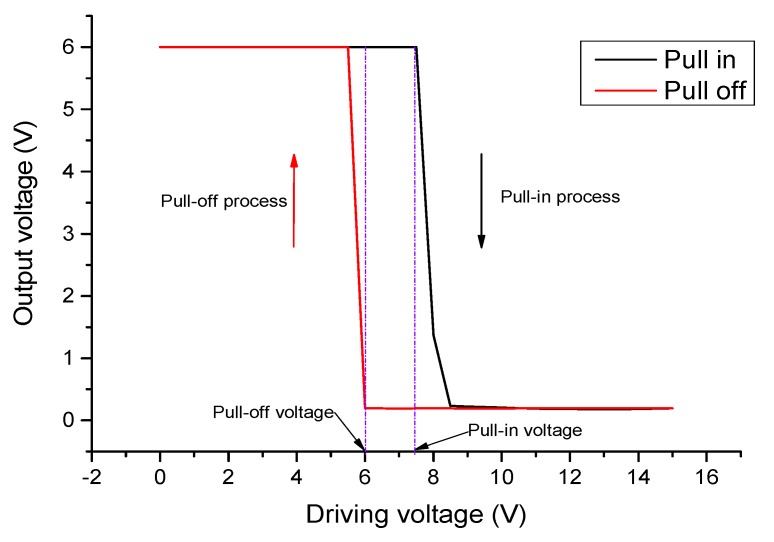
Hysteresis loop of pull-in and pull-off voltage.

Experimental results show that the average pull-in voltages are about 7.5–8.0 V, which is very similar to the simulated voltages of 7.81–8.02 V. The variation among relays with different contact electrode positions are not distinguishable due to the fabrication error. This is comprehensible because the fabrication error is of just 0.1 μm, which has a significant effect on the pull-in voltage with the air gap being designed as 1.5 μm. However, the actuation voltage of 8 V is low enough for applicability. The average pull-off voltages are about 5.5–6.0 V, which are lower than the pull-in voltages. The actuation process of a relay is shown in Figure 9. In this figure, the measured pull-in voltage and pull-off voltages are 7.5 V and 6 V respectively.

### 5.3. Switching-On and Switching-Off Time

**Figure 10:**
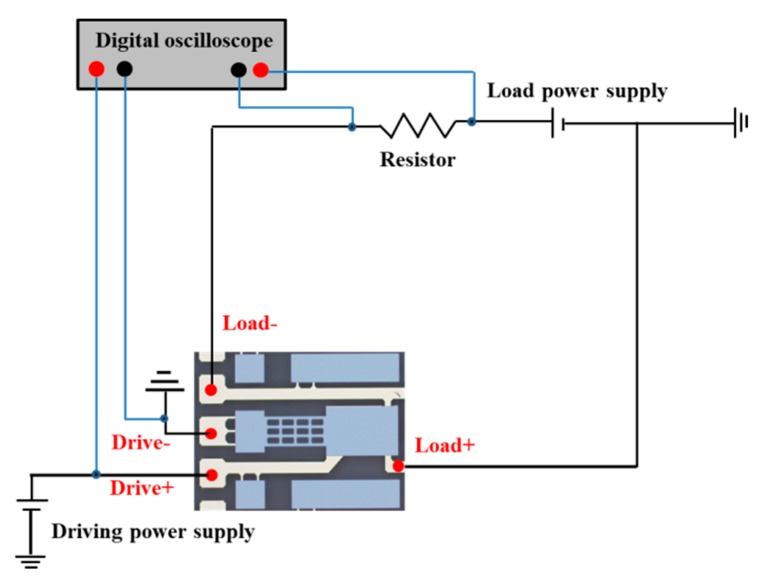
The setup schematic for measuring the switching-on and switching-off time.

Switching-on time refers to the duration from the time when the pull-in voltage of the MEMS relay attained to the time when the state of the proposed relay changed to be ON. Switching-off time refers to the duration from the time when the pull-off voltage of the MEMS relay attained in the pull-off process to the time when the state of the proposed relay changed to be OFF. Figure 10 shows the setup schematic for measuring the switching-on and switching-off time. The driving power supply applies a scanning voltage of 0 to 10 V to the drive terminals and the load power supply voltage set to be 5 V. During the experiment, the digital oscilloscope records the waveform change of voltages. 

**Figure 11:**
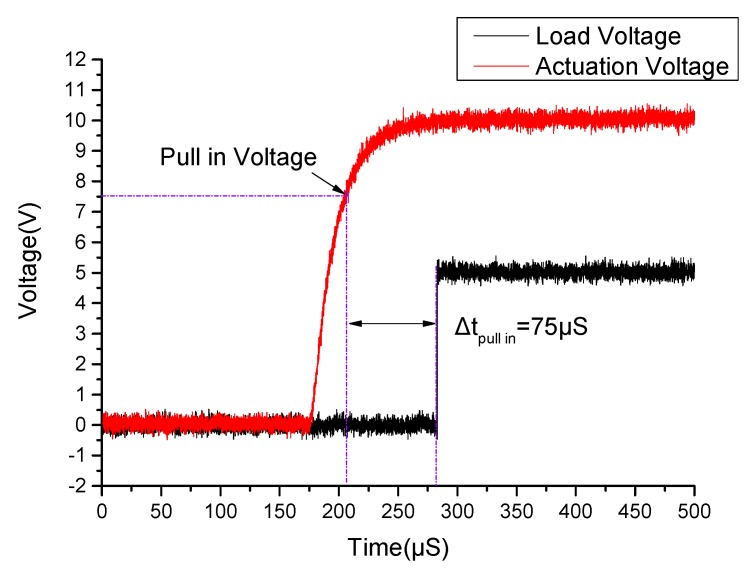
The measured switching-on time of the proposed MEMS relay.

Figure 11 shows the result of the switching-on time measurement experiment. As shown in Figure 11, the measured switching-on time of the proposed relay is about 75 μS. During the pull-in process, when the actuation voltage attains to 7.5 V (pull-in voltage), the driving plate of cantilever begins to bend to the contact electrode. After 75 μS, the circuit gets connected, and the voltage at both ends of the resistor reaches 5 V.

**Figure 12:**
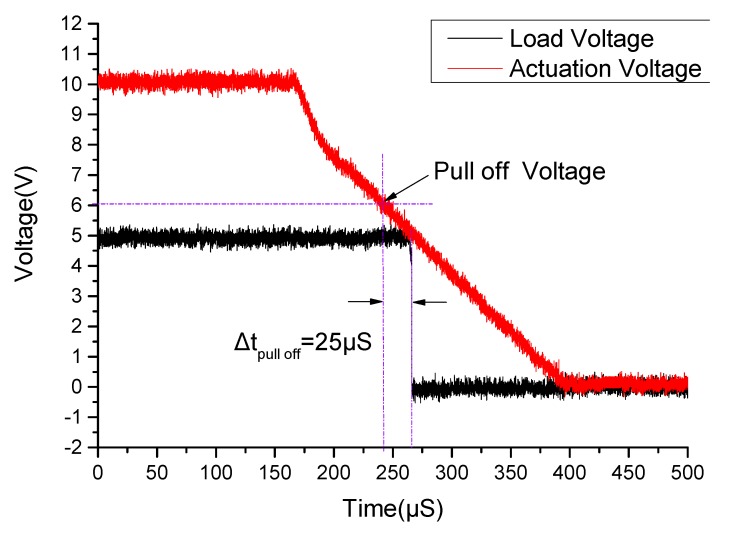
The measured switching-off time of the proposed MEMS relay.

Figure 12 shows the result of the switching-off time measurement experiment. As is shown in Figure 12, the measured switching-off time of the proposed relay is about 25 μS. During the pull-off process, when the actuation voltage drops to 6 V (pull-off voltage), the driving plate of cantilever begins to rebound to balance position. After 25 μS, the circuit gets disconnected, and the voltage at both ends of the resistor turns to 0 V.

### 5.4. Contact Resistance

**Figure 13:**
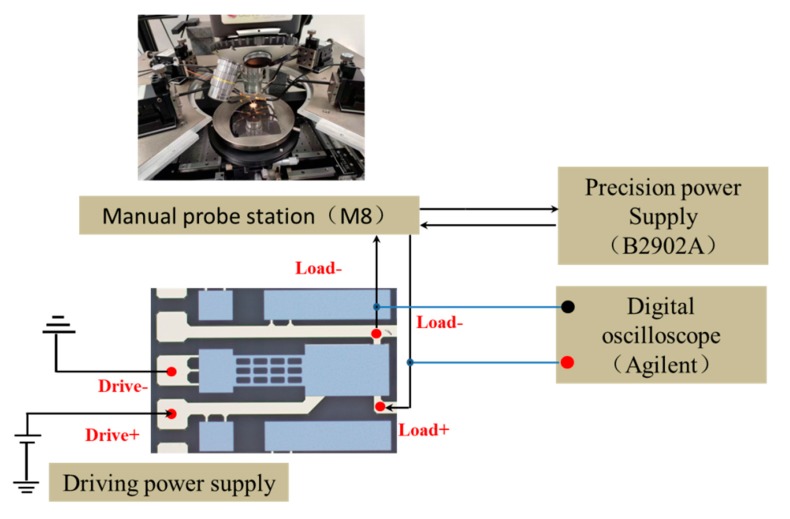
The setup schematic for measuring the contact resistance.

In this research, the contact resistance of the proposed relay is measured using the Kelvin four-wire method. For each test point, there is a constant current source and a voltage detection unit, which are strictly separated, and constitute an independent loop. The voltage line must be connected to a test loop with extremely high input impedance. Meanwhile, the current flowing through the detection line is extremely small, which is approximately zero. The constant current source (B2902A) provides a constant current. The current passes through the contact electrode. Figure 13 shows the setup schematic for measuring the contact resistance. In this figure, the driving power supply provides a constant 10 V voltage to make sure the MEMS relay keeps on. The precision power supply provides a constant current. The digital oscilloscope measures the voltage across the relay. The contact resistance can be expressed as *R* = *V*/*I*.

In order to reduce measurement error, the measurements of the contact resistance were carried out and averaged. Stable and low contact resistance can be obtained when the pull-in voltage is 8 V. The measured contact resistance of each relay was less than 0.4 Ω. The contact resistance was also measured under different load currents. The experimental result shows that the contact resistance was lower at a load current of 20 mA than that at 200 μA. This was due to the softening of the contact asperities, which may result in a more effective contact area; however, it increased slightly at currents higher than 20 mA, which may be caused by the resistivity increasing induced by the high temperature at localized asperities.

### 5.5. Contact Lifetime

**Figure 14:**
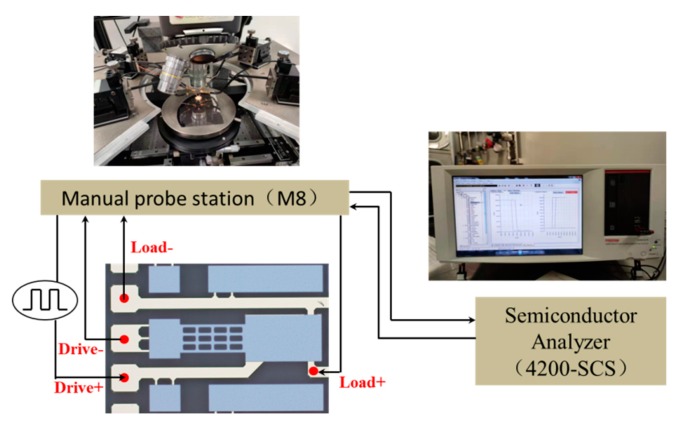
The setup schematic for measuring the contact lifetime.

The contact lifetime was measured on the manual probe station by the semiconductor analyzer (4200-SCS). The source/measure unit provides two independent channels: one channel was used for providing the driving voltage, the other for measuring the load circuit resistance. The driving voltage was set at 15 V which has a low contact resistance and rapid response. MEMS relays are mostly operated in two modes: cold-switching operation and hot-switching operation. Cold-switching refers to relay closure before applying voltage and voltage removal before relay opening, while hot-switching refers to the relay actuation synchronized with the electrical switching. Figure 14 shows the setup schematic for measuring the contact lifetime.

In the cold-switching operation experiment, the load of the semiconductor analyzer was set at 30 mV and 30 μA. After a 5 × 10^6^ test cycle, the contact resistance of the MEMS relay rises to above 10 Ω, which means a failure of the relay. This may be due to the formation of an insulating film on the contact surface. On the other hand, when the load was set at 12 V and 20 mA, the test was carried out about 6000 cycles before the MEMS relay reached failure.

### 5.6. Comparison of the Performance

Table 2 makes a comparison of the performance for MEMS relays reported in the literature and this research. From this table, we can conclude that the proposed MEMS relay in this research achieves a lower actuation voltage (below 8 V) than the MEMS relays reported in the literature; meanwhile, the contact resistance, switching time, and contact lifetime performance show a certain degree of advantage. 

## 6. Conclusions

In this article, we present a novel cantilever MEMS relay with an embedded contact electrode. This embedded contact electrode structure helps to achieve a low actuation voltage (below 8 V) and high device performance simultaneously. To thoroughly investigate and analyze the novel cantilever MEMS relay, a static theoretical model of the structure was developed. Based on the model, the cantilever MEMS relay was designed and optimized. Then, the relays were fabricated by the bulk-silicon micromachining process based on the silicon–glass anodic bonding technology. Finally, the switching performance of the novel cantilever MEMS relay was measured. The experimental results demonstrate that the MEMS relay has a low actuation voltage and high performance.

## Figures and Tables

**Table 1:** Geometric parameters of the proposed MEMS relay.

Symbol	Description of Parameters	Value
*L*_1_	Length of the hollow spring	460 μm
*W*_1_	Equivalent Width of the hollow spring (coupled with four 20 μm wide microbeams)	80 μm
*L*_2_	Length of the driving plate	540 μm
*W*_2_	Width of the driving plate	330 μm
*L*_3_	Length of the contact electrode	50 μm
*W*_3_	Width of the contact electrode	180 μm
*t*	Thickness of the cantilever beam	22 μm
*g*_0_	Distance of the air gap	1.5 μm
*g*_c_	Distance of the contact gap	0.7 μm
x_c_	Position of the contact electrode	0–250 μm

**Table 2:** Comparison of the performance for MEMS relays reported in literature.

Research Institute	Actuation Voltage (V)	Contact Resistance (Ω)	Switching Time (μS)	Contact Lifetime
MIT [16]	20	0.05	20,000–50,000	-
KAIST [17]	40	0.005	230	4.9 × 10^5^
UCSD [18]	75–90	1.5	<10	-
ADI [19]	80	1.6	<30	10 × 10^9^
This research	<8	0.4	<75	5 × 10^6^
